# Drug-induced cell cycle modulation leading to cell-cycle arrest, nuclear mis-segregation, or endoreplication

**DOI:** 10.1186/1471-2121-12-2

**Published:** 2011-01-13

**Authors:** Asako Sakaue-Sawano, Tamiyo Kobayashi, Kenji Ohtawa, Atsushi Miyawaki

**Affiliations:** 1 Life Function and Dynamics, ERATO, JST, 2-1 Hirosawa, Wako-city, Saitama 351-0198, Japan; 2 Laboratory for Cell Function and Dynamics, Advanced Technology Development Group, Brain Science Institute, RIKEN, 2-1 Hirosawa, Wako-city, Saitama 351-0198, Japan; 3 MIS Division, Olympus Corp., 2-3 Kuboyama-cho, Hachioji, Tokyo 192-8512, Japan; 4 Brain Science Research Division, Brain Science and Life Technology, Research Foundation, 1-28-12 Narimasu, Itabashi, Tokyo 175-0094, Japan

## Abstract

**Background:**

Cancer cell responses to chemotherapeutic agents vary, and this may reflect different defects in DNA repair, cell-cycle checkpoints, and apoptosis control. Cytometry analysis only quantifies dye-incorporation to examine DNA content and does not reflect the biological complexity of the cell cycle in drug discovery screens.

**Results:**

Using population and time-lapse imaging analyses of cultured immortalized cells expressing a new version of the fluorescent cell-cycle indicator, Fucci (*F*luorescent *U*biquitination-based *C*ell *C*ycle *I*ndicator), we found great diversity in the cell-cycle alterations induced by two anticancer drugs. When treated with etoposide, an inhibitor of DNA topoisomerase II, HeLa and NMuMG cells halted at the G_2_/M checkpoint. HeLa cells remained there, but NMuMG cells then overrode the checkpoint and underwent nuclear mis-segregation or avoided the checkpoint and entered the endoreplication cycle in a drug concentration dependent manner. In contrast, an inhibitor of Cdk4 led to G_1 _arrest or endoreplication in NMuMG cells depending upon the initial cell-cycle phase of drug exposure.

**Conclusions:**

Drug-induced cell cycle modulation varied not only between different cell types or following treatment with different drugs, but also between cells treated with different concentrations of the same drug or following drug addition during different phases of the cell cycle. By combining cytometry analysis with the Fucci probe, we have developed a novel assay that fully integrates the complexity of cell cycle regulation into drug discovery screens. This assay system will represent a powerful drug-discovery tool for the development of the next generation of anti-cancer therapies.

## Backgrounds

Effective anticancer agents preferentially kill cancer cells, and many anticancer drugs directly induce DNA damage and/or inhibit DNA repair pathways. In normal cells, in response to the DNA damage induced by anticancer drugs, a complex signaling network is activated to prevent the replication of damaged DNA and the transmission of damage-related alterations in DNA sequences to the next generation of cells [1]. In contrast, cancer cells generally have defects in many of these pathways including factors regulating cell-cycle checkpoints. Such cells continue to divide in the face of widespread DNA damage, and this ultimately leads to cancer cell death. However, cancer cell responses to anticancer drugs vary [2,3]. While some of the defects common to cancer cells enhance their sensitivity to drugs, other changes found in malignantly transformed cells increase their chemotherapy resistance. Additionally, environmental factors can affect the cellular response to anticancer drugs. Furthermore, there are several well-described cell-cycle variations that eukaryotic cells can exhibit. One common variant is the endoreplication cycle [4-11], in which cells increase their genomic DNA content without dividing. In order to fully integrate the complexity of the cell cycle into drug discovery screens, it is imperative to take a multifaceted approach, combining conventional cytometry analysis with a new technique that allows for visualizing the cell cycle progression of individual cells in real time.

Cell cycle progression is dependent upon the coordinated regulation of ubiquitination, and we harnessed this system to develop a genetically encoded indicator of cell cycle progression: Fucci (*F*luorescent *U*biquitination-based *C*ell *C*ycle *I*ndicator) [12]. The original Fucci probe was generated by fusing mKO2 (monomeric Kusabira Orange2) and mAG (monomeric Azami Green) to the ubiquitination domains of human Cdt1 and Geminin, respectively. These two chimeric proteins, mKO2-hCdt1(30/120) and mAG-hGem(1/110), accumulate reciprocally in the nuclei of transfected cells during the cell cycle, labeling the nuclei of G_1 _phase cells orange and those of cells in S/G_2_/M phase green. Thus, they function as G_1 _and S/G_2_/M markers, respectively. We previously injected HeLa and NMuMG (normal murine mammary gland) cells constitutively expressing Fucci into the mammary fat pad of nude mice, to monitor changes in the cell cycle profiles of the foreign cells [12]. Interestingly, while HeLa/Fucci cells replicated and began to spread metastatically, NMuMG/Fucci cells stopped proliferating.

**Figure 1:**
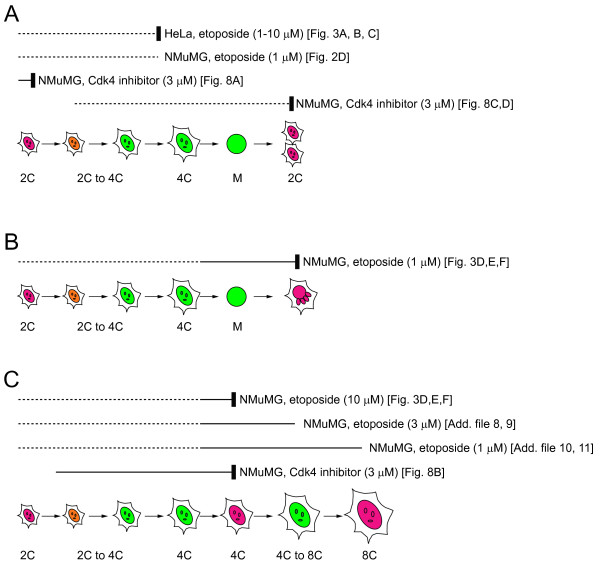
**Schemes illustrating the cell cycle alteration(s) observed in HeLa or NMuMG cells treated with different concentrations of etoposide or Cdk4 inhibitor**. The cell-cycle processes that were visualized in this study are indicated by solid lines. Cell-cycle arrest (retardation) or nuclear mis-segregation is indicated by a vertical thick bar. C values denote DNA content as a multiple of the normal haploid genome. (A) Cell cycle arrest. (B) Nuclear mis-segregation. (C) Endoreplication cycle. Figures depicting the primary data are indicated in the square brackets. Nuclei are colored according to Fucci2 signals.

In the present study, we developed new Fucci constructs with different fluorescent proteins, and we then generated stable transformants of HeLa and NMuMG cells with these constructs. We used these newly generated cell lines as an *in culture *means for examining the impact of anticancer drugs on the cell cycle. We observed a much greater variety of drug-induced cell cycle variations than expected, as schematized in Figure 1, suggesting the need to evaluate the effects of anticancer therapies under a variety of circumstances. Our assay system will be particularly relevant for the development of novel anti-cancer pharmaceuticals.

## Results

### Generation of Fucci2 constructs

We wished to develop new Fucci derivatives with different fluorescent properties, and, towards that end, we used a yellowish green monomeric variant of *Aequorea *green fluorescent protein (GFP) (mVenus) [13,14] and a red monomeric fluorescent protein (mCherry) [15]. mCherry and mVenus were substituted for mKO2 and mAG in the original Fucci constructs to generate mCherry-hCdt1(30/120) and mVenus-hGem(1/110), respectively (Additional file 1A). The new Fucci derivative was named "Fucci2." This particular combination of fluorophores provides better color contrast than that of Fucci (Additional file 1B). Additionally, the fluorescent characteristics of the widely used fluorophore enhanced GFP (EGFP) are able to be spectrally distinguished from those of Fucci2. This will facilitate imaging experiments where cell cycle progression is monitored in parallel with protein subcellular localization and/or signaling events.

To generate cell lines stably expressing Fucci2, lentiviral vectors were used for co-transduction of mCherry-hCdt1(30/120) and mVenus-hGem(1/110) in HeLa cells (HeLa/Fucci2). The mVenus fluorescence disappeared rapidly in late M phase and the red fluorescence of mCherry became detectable in early G_1 _phase; during the G_1_/S transition both fluorophores were present generating orange fluorescence (Additional files 1C and 2). HeLa/Fucci2 cells were divided into red-, orange-, and yellowish green-emitting populations (Additional file 1D), and their DNA contents were analyzed following Hoechst33342 staining. Yellowish green and orange cells had fully- and partially-replicated complements of DNA, respectively (Additional file 1E). We have also made a stable transformant constitutively expressing Fucci2 using normal murine mammary gland (NMuMG) cells (NMuMG/Fucci2) (Additional file 3).

To analyze cell cycle progression *in culture*, we used two complementary approaches. First, we collected a large number of cells that had been treated with varying concentrations of drug for different periods of time, and we stained their nuclei with Hoechst 33342 or 4',6-diamidino-2-phenylindole (DAPI). The number of cells in G_1_, G_1_/S, and S/G_2_/M phases were counted, and we quantified the DNA content of individual cells. For these large-scale analyses we used a fluorescence-activated cell sorter (FACS) and CELAVIEW RS100 (Olympus). The latter is a drug-screening platform designed for fully automated image acquisition and data analysis [16,17]. In addition to cytometry functions for analyzing large data sets, the CELAVIEW system links acquired images with relevant cellular and biochemical information [18-20]. Alternatively, we used time-lapse imaging to monitor a relatively small number of live cells using an LCV100 (Olympus), a computer-assisted fluorescence microscopy system, which can monitor up to 8 cell culture dishes containing different concentrations of drugs under stabilized conditions over an extended period of time.

### Etoposide

**Figure 2:**
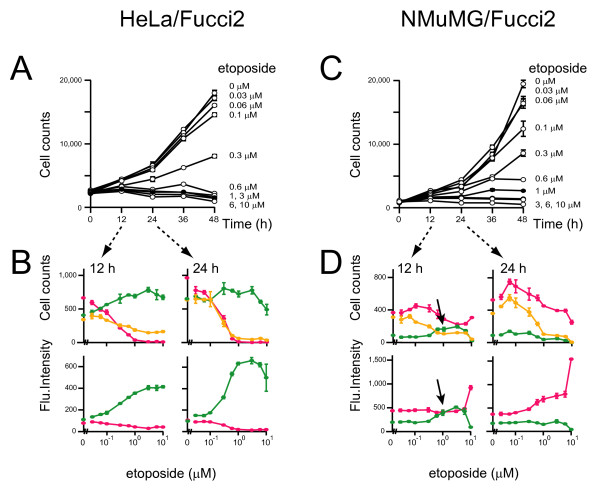
**Proliferation of cultured tumor cells at various concentrations of etoposide**. HeLa/Fucci2 (A and B) or NMuMG/Fucci2 (C and D) cells were plated at the same density in multiple wells and grown in the presence of the indicated concentrations of etoposide. Following a culture period of 0, 12, 24, 36, or 48 h, cells were fixed, and total cell numbers were counted after nuclear staining with DAPI to generate cell proliferation curves (A and C). Additionally, the number of red-, orange-, and yellowish green-emitting cell populations as well as the average fluorescence intensities of individual nuclei were automatically counted. The cell cycle profiling data with various concentrations of the compound at 12 and 24 h are shown with the Fucci colors (B and D). Because Fucci probes utilize protein degradation mechanisms and reflect accumulation of Cdt1 and Geminin, their signal intensities (the average fluorescence intensities of individual nuclei) depend on the duration of cell-cycle phases. The prolongation of the G_2 _phase in the presence of 1 μM etoposide is indicated by black arrows. Data points are means ± SD of triplicate samples (wells).

**Figure 3:**
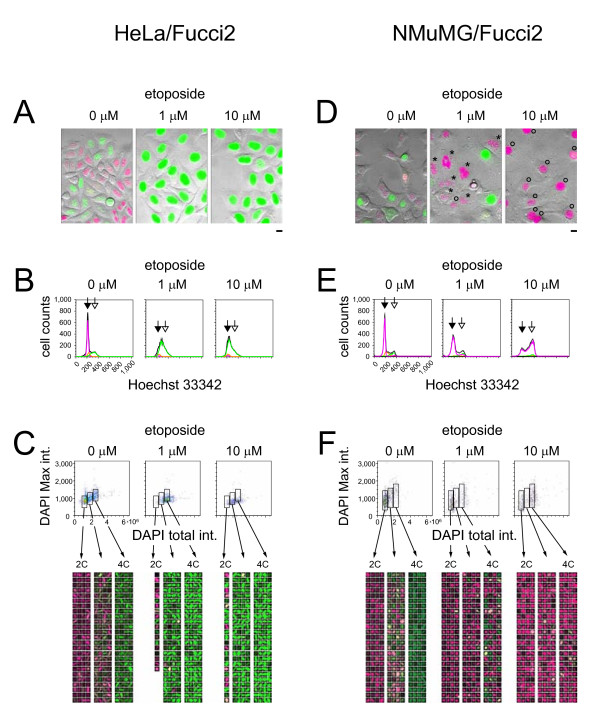
**Cell cycle profiles of cultured tumor cells treated with etoposide**. HeLa/Fucci2 cells (A, B, and C) and NMuMG/Fucci2 cells (D, E, and F) were treated with 0 μM, 1 μM, and 10 μM etoposide (left, middle, and right of each panel, respectively) for 24 h. (A and D) Typical fluorescence images of cells acquired by LCV100 are shown. Scale bar, 10 μm. (D) The red nuclei with chromosome fragmentation and 4C DNA content are labeled with asterisks and open circles, respectively. (B and E) Cell cycle profiles obtained by FACS. Cell cycle phases highlighted by fluorescence are colored. For measuring DNA content, cells were stained with Hoechst33342. Solid and open arrows indicate the 2C and 4C DNA containing cells, respectively. (C and F) Cell cycle profiles (two-dimensional scatter plots and gallery images) obtained by CELAVIEW. For measuring DNA content, cells were stained with DAPI. High-magnification images of the gallery are presented in Figure 4A (HeLa/Fucci2, 0 μM etoposide, for Figure 3C left), Figure 4B (HeLa/Fucci2, 1 μM etoposide, for Figure 3C middle), Figure 4C (HeLa/Fucci2, 10 μM etoposide, for Figure 3C right), Figure 5A (NMuMG/Fucci2, 0 μM etoposide, for Figure 3F left), Figure 5B (NMuMG/Fucci2, 1 μM etoposide, for Figure 3F middle), and Figure 5C (NMuMG/Fucci2, 10 μM etoposide, for Figure 3F right).

**Figure 4:**
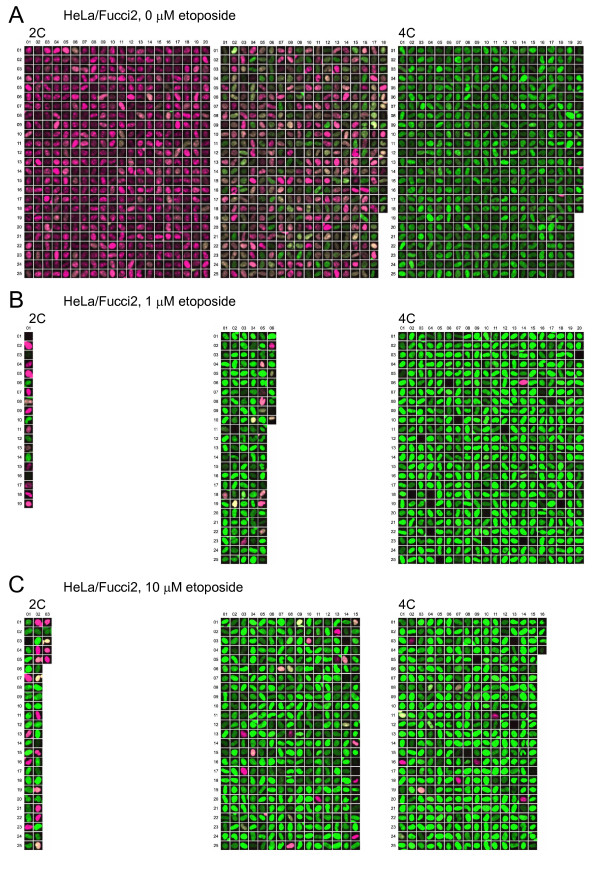
**High-Resolution Gallery Images of HeLa/Fucci2 Cells**. High-magnification images of the gallery are presented. (A) HeLa/Fucci2 cells showing normal mitotic cycling. (B) HeLa/Fucci2 cells treated with 1 μM etoposide. (C) HeLa/Fucci2 cells treated with 10 μM etoposide. The numbers of images indicate the size of individual data populations (Figure 3C). 2C, diploid; 4C, tetraploid.

Etoposide has been used as an anticancer agent for some time, and it acts by inhibiting DNA topoisomerase II, a key enzyme involved in DNA decatenation [21,22]. The action of etoposide is greatest at the G_2_/M checkpoint, and we used cells expressing Fucci2 to monitor the effects of etoposide exposure. Using CELAVIEW we demonstrated that proliferation of HeLa/Fucci2 cells was blocked by >0.1 μM of etoposide (Figures 2A and 2B). The growth arrest was associated with a substantial loss of red- and orange-emitting cell populations. As a result, only cells exhibiting bright yellowish green nuclei were observed after a 24 h treatment with 1 μM or 10 μM etoposide (Figures 2B and 3A). The accumulation of non-diploid (>2C) cells with yellowish green nuclei was verified using FACS (Figure 3B), CELAVIEW (Figures 3C and 4), and time-lapse imaging by LCV100 (Additional file 4). These results indicate that etoposide at concentrations >0.1 μM induced cell cycle arrest at the G_2 _DNA checkpoint in HeLa/Fucci2 cells (Figure 1A).

**Figure 5:**
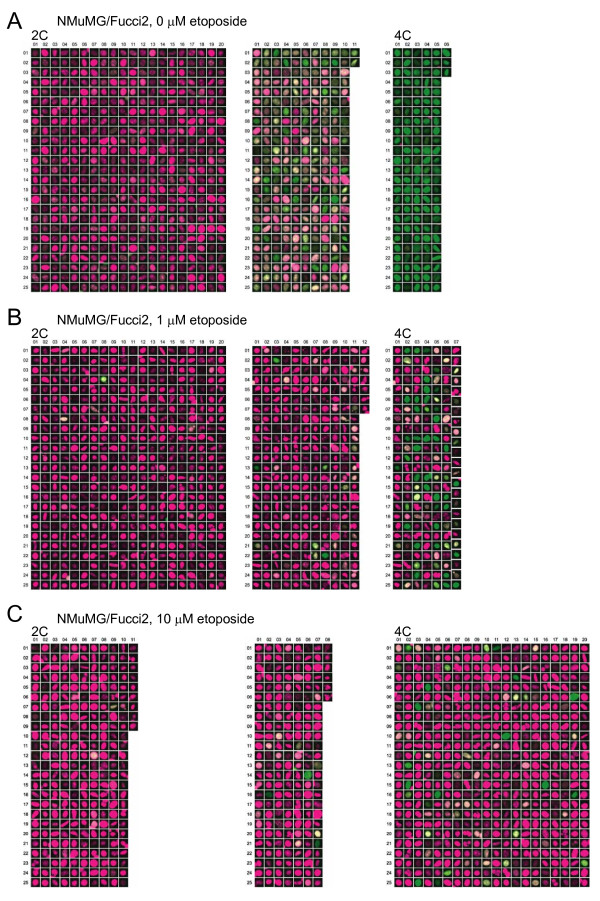
**High-Resolution Gallery Images of NMuMG/Fucci2 Cells**. High-magnification images of the gallery are presented. (A) NMuMG/Fucci2 cells showing normal mitotic cycling. (B) NMuMG/Fucci2 cells treated with 1 μM etoposide. (C) NMuMG/Fucci2 cells treated with 10 μM etoposide. The numbers of images indicate the size of individual data populations (Figure 3). 2C, diploid; 4C, tetraploid.

The cell cycle of NMuMG/Fucci2 cells was also affected by concentrations of etoposide >0.1 μM (Figure 2C), but the cellular response to drug was much more variable. Time-lapse imaging experiments fully traced the cell cycle progression of individual cells in the absence and presence of 1 μM etoposide (Additional files 3 and 5, respectively). These cells underwent transient G_2 _arrest, and the prolongation of the G_2 _phase was evident in population analysis (Figure 2D, black arrows). The cells then entered M phase and underwent "nuclear mis-segregation" [23-27]. This is characterized by nuclear envelope breakdown and chromosome condensation, but failed mitosis and subsequent chromosome fragmentation (Figure 1B). In fact, there was a marked accumulation of cells with fragmented, red nuclei at 24 h (Figure 3D, 3F, each middle; Figure 5B).

In contrast, treatment of cells with 10 μM etoposide for 24 h produced a substantial number of NMuMG/Fucci2 cells with large, clear, and red nuclei (Figure 3D, right) rather than fragmented nuclei. We initially hypothesized that these cells had undergone G_1 _cell cycle arrest, but analysis of cells by FACS demonstrated a large population of cells with red nuclei with double the DNA content of normal cells (4C) (Figure 3E, right, an open arrow). Additionally, by time-lapse imaging we frequently observed NMuMG/Fucci2 cell nuclei progress from yellowish green to red without undergoing cell division (Additional file 6). No cellular features consistent with M phase were seen, including nuclear envelope breakdown and chromosome condensation, during the yellowish green-to-red color conversion. The presence of a large population of tetraploid cells suggests a transition from the mitotic to endoreplication cycles (Figure 1C), characterized by increases in cellular DNA content achieved by bypassing mitosis and cytokinesis. Gallery views of images of three data populations with varied DNA content are shown in the two-dimensional scatter plots created by CELAVIEW (Figure 3F, right and Figure 5C), demonstrating that each cell carrying the large red nucleus was tetraploid. We next monitored NMuMG/Fucci2 cells for an extended time period up to 96 h in the presence of 10 μM etoposide. During the period, most cells underwent only one round of DNA endoreplication generating large red nuclei, confirming that cells remained tetraploid at the end of the observation period (96 h).

**Figure 6:**
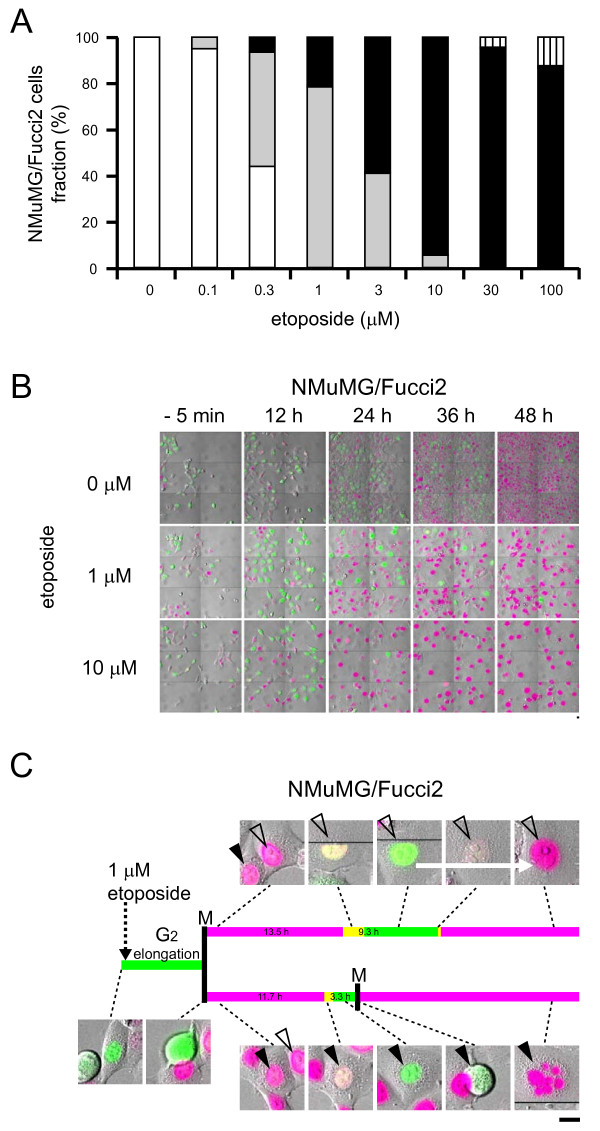
**Responses of NMuMG/Fucci2 cells to etoposide analyzed using the LCV100**. (A) Histogram (fraction %) of occurrence of normal cycling or G_2 _arrest (white), nuclear mis-segregation characterized by chromosome fragmentation (gray), tetraploid cells generated by endoreplication (black), and cell death (vertical line) at various concentrations of the drug. (B) Fluorescence images of NMuMG/Fucci2 cells treated with 0, 1, and 10 μM etoposide. In the presence of etoposide, the yellowish green nuclei predominated transiently at 12 h, suggesting cell cycle arrest at the G_2_/M checkpoint. Scale bars, 10 μm. (C)In the presence of 1 μM etoposide, an NMuMG/Fucci2 cell divided after a relatively long stay in G2 phase. The two daughter cells experienced different fates; one cell (labeled with an open arrowhead) underwent endoreplication (top), and the other (labeled with a solid arrowhead) underwent nuclear mis-segregation (bottom). The yellowish green-to-red color conversion is indicated by an white arrow (top). Scale bar, 10 μm.

We further examined cell cycle modulation at various concentrations of etoposide (Figures 6A, B and Additional file 7). We monitored cells for 48 h with time-lapse imaging, and we quantified the incidence of normal cycling or G_2 _arrest, nuclear mis-segregation characterized by chromosome fragmentation, and tetraploid cells generated by endoreplication. While nuclear mis-segregation was common in cells treated with 0.3 - 3 μM etoposide, tetraploid cells appeared at concentrations ≥1 μM.

### Cdk4 inhibitor

Although etoposide predominantly affects the G_2 _to M transition, we wished to examine an additional anti-cancer therapeutic. We selected the cell-permeable Cdk4 inhibitor (2-bromo-12,13-dihydro-5H-indolo[2,3-a]pyrrolo[3,4-c]carbazole-5,7(6H)-dione) [28] for further study with cells expressing Fucci2. This drug inhibits the growth of tumor cells (HCT-116 and NCI-H460 cells) with an IC_50 _<3.0 μM by blocking Rb phosphorylation and inducing G_1 _cell cycle arrest.

**Figure 7:**
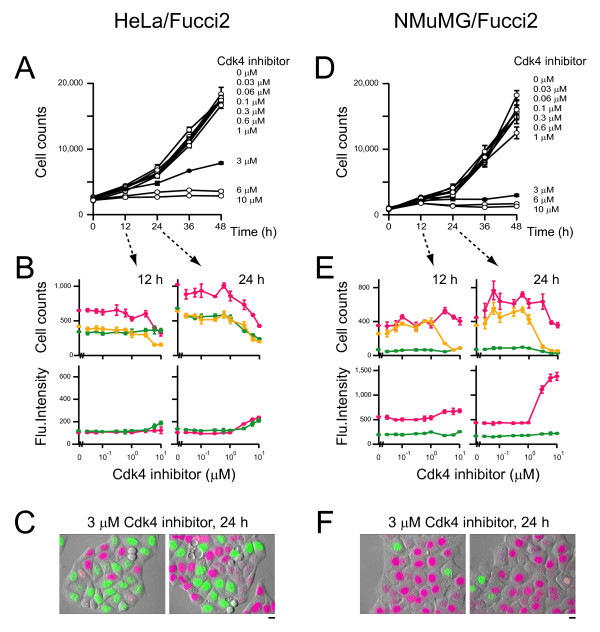
**Proliferation of cultured tumor cells at various concentrations of Cdk4 inhibitor**. HeLa/Fucci2 (A, B, and C) or NMuMG/Fucci2 (D, E, and F) cells were plated at the same density in multiple wells and grown in the presence of various concentrations of Cdk4 inhibitor. Following culture period of 0, 12, 24, 36, or 48 h, cells were fixed, and total cell numbers were counted after nuclear staining with DAPI to draw cell proliferation curves (A and D). In addition, the number of red-, orange-, and yellowish green-emitting cells were determined and the averaged fluorescence intensities of individual nuclei were counted. The cell cycle profiling data with various concentrations of the compound at 12 and 24 h are shown (B and E). Data points are means ± SD of triplicate samples (wells). Two representative fluorescence images of HeLa/Fucci2 (C) and NMuMG/Fucci2 (F) cells that were treated with 3 μM Cdk4 inhibitor for 24 h are shown. Scale bar, 10 μm.

Treatment of HeLa/Fucci2 cells with the inhibitor significantly inhibited cell proliferation in a dose-dependent manner (Figure 7A). Interestingly, however, this did not alter the fractions of red-, orange-, and yellowish-green-emitting populations (Figure 7B, top), but there was an increase in all Fucci signals in individual nuclei (Figure 7B, bottom), indicating a slow but continuous cycling of HeLa/Fucci2 cells. Additionally, we identified HeLa/Fucci2 cells in G_1_, S, G_2_, and M phases after treatment with 3 μM Cdk4 inhibitor for 24 h by time-lapse imaging (Figure 7C).

**Figure 8:**
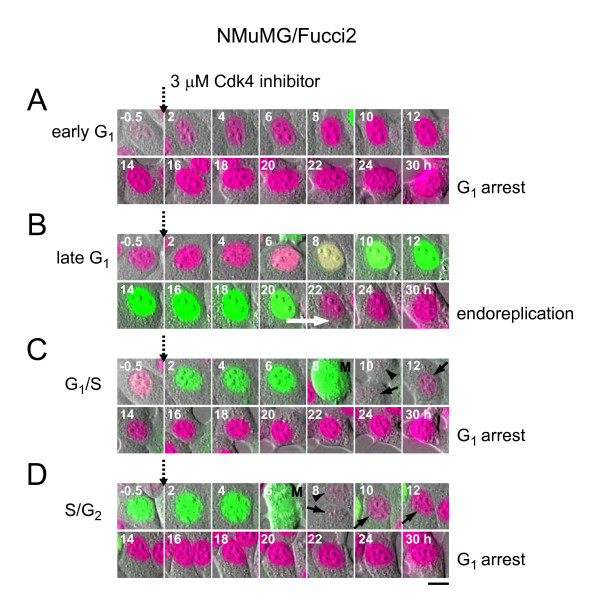
**Exposure to 3 μM Cdk4 inhibitor caused NMuMG/Fucci2 cells to undergo G**_**1 **_**arrest or endoreplication in a cell-cycle phase dependent manner**. When the drug was applied, the observed cells were in early G_1 _(A), late G_1 _(B), G_1_/S (C), and S/G_2 _(D) phases. Scale bar, 10 μm. (B) A white arrow indicates the endoreplication. (C and D) M, M phase. A solid arrow indicates a daughter cell, which was tracked. A solid arrowhead indicates the other daughter cell.

The cell cycle of NMuMG/Fucci2 cells was affected by >1 μM of the Cdk4 inhibitor, giving similar cell proliferation curves (Figure 7D) to those of HeLa/Fucci2 cells (Figure 7A). However, the cell cycle profile data differed between the two cell lines. After 24 h, the cell populations in G_1_/S and S/G_2_/M phases were nearly absent, while that in G_1 _phase was retained (Figure 7E, top) with increased fluorescence signals (Figure 7E, bottom). Exposure to the compound (>1 μM) for 24 h resulted in G_1 _cell cycle arrest as shown in two pictures (Figure 7F). Time-lapse imaging showed that most of the cells treated with 3 μM Cdk4 inhibitor underwent G_1 _arrest directly or after single cell divisions during a period of 30 h (Figures 1A and 8A, 8C, 8D). Notably, tetraploid cells generated by endoreplication was also observed in the NMuMG/Fucci2 cells that had been in late G_1 _phase when initially exposed to drug (Figures 1C and 8B).

## Discussion

Tumor cells stably transfected with fluorescent proteins enable scientists to visualize many important aspects of cancer in real time at the single cell level. For example, transfected tumor cells have been visualized either through surgically created chronic-transparent windows or directly through the opened skin of living animals [29]. This intravital imaging provides a powerful tool for observing cancer initiation and progression and evaluating the efficacy of candidate cancer drugs in vivo. On the other hand, assays using tumor cells grown in culture provide reliable information about cancer mechanisms, and are amenable to automated high-throughput screening [16-20]. Using a modified fluorescent indicator of cell cycle progression (Fucci2) and cultured immortalized cells, we investigated the mechanism(s) by which anticancer drugs modulate the cell cycle. While population analysis provided statistical data, time-lapse high-resolution imaging analysis allowed us to explore the processes of cell cycle alteration in great detail. Drug-induced cell cycle modulation varied between different cell types or following treatment with different drugs, but, intriguingly, differences were observed between cells treated with different concentrations of the same drug or following drug addition during different phases of the cell cycle (Figure 1).

Three different fates await NMuMG/Fucci2 cells at the G_2_/M checkpoint after exposure to etoposide. At low concentrations of the drug, cells halt their cell cycle at the checkpoint. At intermediate concentrations, cells override the checkpoint and undergo nuclear mis-segregation. At high concentrations, cells avoid the checkpoint and enter the endoreplication cycle. Endoreplication may render cells resistant to conditions that induce DNA damage, possibly because they have several copies of each gene and do not need to segregate their chromosomes [30]. From a clinical perspective, the tendency of high concentrations of etoposide to induce endoreplication in NMuMG cells may provide insight into the persistence of disease and development of chemotherapy resistance.

When cells are treated with drugs that affect the cell cycle, drug concentration is an important parameter affecting the cellular response. However, other factors including environmental factors may be critical. A previous study showed that hematopoietic cells treated with etoposide halt their cell cycle at G_2_/M or experience nuclear mis-segregation in the presence or absence of hematopoietic cytokines, respectively [22]. Also, asymmetric cell division may generate different vulnerabilities to drugs. We observed that cell division produced two daughter cells with different fates in the presence of 1 μM etoposide; one underwent nuclear mis-segregation and the other entered the endoreplication cycle (Figure 6C). This observation excludes the possibility that the diversity of drug response results from inherent heterogeneity of the cell lines.

Mammalian megakaryocytes and trophoblast giant cells undergo endoreplication to reach DNA ploidies with a DNA content >1,000-fold higher than that of a normal diploid cell [4,5,7-9]. We isolated megakaryocytes and trophoblast giant cells from Fucci transgenic mouse lines, and successfully imaged the endoreplication cycles in these cell types cultured on a coverslip (A. S.-S, and A. M. unpublished results); the nuclei changed color alternately many times between green and red while enlarging. This observation verifies that oscillations in ubiquitination involving the SCF^Skp2 ^and APC^Cdh1 ^complexes function during the endoreplication cycle, and Fucci technology allows for the visualization of endoreplication [31-34]. In contrast to the developmentally programmed endoreplication [9], most of the NMuMG/Fucci2 cells treated with etoposide experienced endoreplication only once, stably remaining tetraploid during our observation period of ≤4 days. Although it is known that anticancer drugs induce endoreplication to generate polyploidy in a variety of cell types, the predominant accumulation of tetraploid cells was also reported to result from inhibition of DNA topoisomerase II in other biological systems [21]. Conventional cell cycle analysis that only quantifies dye-incorporation using FACS may misinterpret this long-lasting tetraploid state as G_2 _arrest. The information provided by the Fucci probes regarding the cell cycle phase allowed us to identify the single-round of endoreplication in these cells. Although a single round of endoreplication was the principal outcome in this study, a small fraction of the NMuMG cells was observed to go through multiple endoreplication cycles in the presence of 1 - 3 μM etoposide (Figure 1C, Additional files 8 - 11).

Fucci allows for visualizing cell-cycle progression in real time at the single cell level. Combined with information of DNA content obtained by cytometry analysis, Fucci technology can reveal complex aspects of drug-induced cell cycle alteration(s). Importantly, the technology becomes more powerful with long-term time-lapse imaging experiments. This dynamic data will advance our understanding of how individual cells respond to a drug. In our time-lapse imaging experiments where NMuMG/Fucci2 cells were continuously treated with 1 - 3 μM etoposide, for example, nuclear mis-segregation and endoreplication were mutually exclusive; we did not see any transition from one to the other. Although it was reported that chromosomes were condensed during formation of endopolyploid cells [4,24], the endoreplication observed in the present study lacked all vestiges of mitosis, including chromosome condensation, nuclear envelope breakdown, and the reorganization of microtubules that builds the spindle. The other finding made by time-lapse imaging was that NMuMG/Fucci2 cells entered the endoreplication cycle if they had been in late G_1 _phase when the Cdk4 inhibitor was added to the medium; otherwise, the cells exposed to the drug showed G_1 _cell-cycle arrest. These features could not be elucidated by conventional cell cycle analysis using fixed cell samples.

Fluorescence imaging of stably transformed Fucci2-expressing cells in culture will provide reliable pharmaco-dynamic readouts for the proliferation and death of cancer cells. Through full integration of statistical and image data, our multifaceted assay system represents a validated strategy for characterization of drugs that modulate the cell cycle for anticancer drug screening and for cell toxicity studies.

## Conclusions

A complete understanding of the factors regulating cell cycle progression is essential for the generation of new anti-cancer agents, but the information gained from using existing reagents is becoming increasingly limited. We recently developed a novel genetically encoded indicator of cell cycle progression, Fucci (Fluorescent Ubiquitination-based Cell Cycle Indicator) that exploits the regulation of cell cycle dependent ubiquitination. In the Fucci technique, cells are genetically modified to express the G_1 _marker Cdt1 and the S/G_2_/M marker Geminin fused to orange and green fluorescent tags, respectively. As a result, actively replicating cell nuclei in S/G_2_/M phases exhibit green fluorescence, while nuclei that are in G_1 _and not yet actively dividing fluoresce orange. In this paper, we describe a new Fucci derivative, Fucci2, which utilizes red and yellowish green fluorescent tags. Using cultured immortalized cells (HeLa and NMuMG cells) that constitutively express Fucci2, we identify several possibly significant variations in the cell cycle that cells exposed to anticancer drugs (inhibitors of DNA topoisomerase II or Cdk4) can undergo. Interestingly, Drug-induced cell cycle modulation varied not only between different cell types or following treatment with different drugs, but also between cells treated with different concentrations of the same drug or following drug addition during different phases of the cell cycle. Importantly, these findings would be missed by conventional cytometry analysis that only quantifies dye-incorporation to examine DNA content. By combining cytometry analysis with the Fucci2 probe, we have developed a novel assay that fully integrates the complexity of cell cycle regulation into drug discovery screens. This assay system will represent a powerful drug-discovery tool for the development of the next generation of anti-cancer therapies.

## Methods

### Gene Construction

cDNA encoding mCherry (kindly provided by Dr. R.Y. Tsien) [15] and mVenus [13] were amplified using primers containing 5'-EcoRI and 3'-NotI sites, and digested products were substituted for the mKO2 gene in mKO2-hCdt1(30/120) [DDBJ/EMBL/GenBank, AB370332] in pCSII-EF vector [35] and the mAG gene in mAG-hGem(1/110) [DDBJ/EMBL/GenBank, AB370333] in pCSII-EF vector, respectively. The sequences reported in this paper have been deposited in the DDBJ/EMBL/GenBank data-base [AB512478, mCherry-hCdt1(30/120); AB512479, mVenus-hGeminin(1/110)].

### Cell Culture

HeLa cells (a subclone of HeLa.S3 cells provided by Dr. Mikoshiba) were grown in DMEM supplemented with 10% fetal bovine serum and penicillin/streptomycin. Mouse NMuMG breast epithelial cells (ATCC; CRL-1636) were grown in DMEM (High glucose) supplemented with 10% fetal bovine serum, penicillin/streptomycin, and 10 μg/ml of insulin (Sigma: I0516). Etoposide (Cat. E1383) and Cdk4 inhibitor (Cat. 219476) were purchased from Sigma and Calbiochem, respectively.

### Lentivirus Production

Replication-defective, self-inactivating lentiviral vectors were used [35]. The CSII-EF-MCS vector encoding mCherry-hCdt1(30/120) or mVenus-hGem(1/110) was co-transfected with the packaging plasmid (pCAG-HIVgp), and the VSV-G- and Rev-expressing plasmid (pCMV-VSV-G-RSV-Rev) into 293T cells. High-titer viral solutions were prepared and used for transduction into HeLa cells or NMuMG cells. Stable transformants were established by diluting cells by single-cell cloning or by FACS.

### Flow Cytometry

Hoechst 33342 solution (56 μl of 1 mg/ml stock) (DOJINDO, Kumamoto, Japan) was added to a 10-cm dish containing HeLa/Fucci2 or NMuMG/Fucci2 cells. After incubation for 30 min, cells were harvested and analyzed using a FACSAria II (BD Bioscience, San Jose, CA). mVenus was excited by a 488-nm laser line (laser diode) and its emission was collected through 515/20BP; mCherry was excited by a 532-nm laser line (diode-pumped solid state laser) and its emission was collected through 610/20 BP. Hoechst 33342 was excited by a UV Laser at 355 nm, and its emission was collected through 405/20 BP. The data were analyzed using FlowJo software (Tree Star).

### Time-Lapse Imaging Cultured Cells

Cells stably expressing Fucci2: mCherry-hCdt1(30/120) and mVenus-hGem(1/110) were grown on 35-mm glass-bottom dishes in phenol red-free Dulbecco's modified Eagle's medium containing 10% fetal bovine serum (FBS). Cells were subjected to long-term, time-lapse imaging using a computer-assisted fluorescence microscope (Olympus, LCV100) equipped with an objective lens (Olympus, UAPO 40X/340 N.A. = 0.90), a halogen lamp, a red LED (620 nm), a CCD camera (Olympus, DP30), differential interference contrast (DIC) optical components, and interference filters. For fluorescence imaging, the halogen lamp was used with a 500AF25 excitation filter, a 525DRLP dichroic mirror, and a 545AF35 emission filter for observing mVenus, and a 565WB20 excitation filter, a 595DRLP dichroic mirror, and a 635DF55 emission filter for observing mCherry. For DIC imaging, the red LED was used with a filter cube containing an analyzer. As the components of the Fucci system are not bound tightly to chromatin, they are detached from dying cells as all dying cell membranes become permeable; we counted the number of cells lacking Fucci signals for measuring cell death. Image acquisition and analysis were performed using MetaMorph 6.37 and 7.6.0.0 software (Universal Imaging, Media, PA), respectively. Movies were assembled using Quick Time software.

### Celaview

HeLa/Fucci2 and NMuMG/Fucci2 cells were plated in 96-well polystyrene plates (Corning, Cat. 3596) at 1,200 cells/100 μl/well and 1,000 cells/100 μl/well, respectively, and grown in a CO_2 _incubator. 24 h later, the medium was changed to 150 μl of fresh medium containing 0 - 10 μM of etoposide or Cdk4 inhibitor. The plates were then incubated for 0, 12, 36 or 48 h. DMSO (0.1%) was used as a vehicle. Cells were washed with phosphate-buffered saline without divalent cations (PBS(-)), fixed with 100 μl of 4% paraformaldehyde in PBS(-) for 20 min at room temperature, stained with 1 μg/ml of DAPI (Roche: Cat. 10236276001) for 10 min at room temperature, and washed with PBS(-) twice. The wells were filled with 150 μl of PBS for observation using the CELAVIEW (OLYMPUS: RS100) instrument, which can scan multiple fields of multiple wells in a plate, and quantitatively analyze each cell in the images by extracting information on the spatial distribution of fluorescently labeled components. DAPI, mVenus, and mCherry were fluorescently detected with U-MNUA2 (OLYMPUS, ex: BP360-370, em: BA420-460), YFP-2427A (Semrock, ex: FF01-500/24, em: FF01-542/27) and U-MWIY2 (OLYMPUS, ex: BP545-580, em: BA610IF), respectively. Fifty-six images at different locations in each well were acquired using a CCD camera (ORCA-AG, Hamamatsu Photonics) through LUCPLFLN20X (OLYMPUS) objective lens.

### Distribution of materials

Stable cell lines; HeLa/Fucci2 and NMuMG/Fucci2 cells will be distributed from the RIKEN BioResource Center Cell Bank http://www.brc.riken.go.jp/lab/cell/english/.

## Authors' contributions

AS-S conceived and designed the study, and performed experiments. TK carried out the imaging experiments using CELAVIEW. KO carried out the FACS analyses. AM conceived and designed the study, and drafted the manuscript. All authors read and approved the final manuscript.

## Supplementary Material

Additional file 1 **Construction of Fucci2**. (A)Constructs with concatenated mCherry and mVenus fused to deletion mutants of human Cdt1 and Geminin. cyan box, Cy motif; pink box, D (destruction) box; black box, NLS (nuclear localization signal). (B) Excitation (broken line) and emission (solid line) spectra of mVenus and mCherry. (C) Fucci2 labels individual G_1 _phase nuclei in red and S/G_2_/M phase nuclei yellowish green. (D,E) Characterization of HeLa/Fucci2 cells. Cells showing red [mCherry(+)mVenus(-)], orange [mCherry(+)mVenus(+)], and yellowish green [mCherry(-)mVenus(+)] fluorescence were collected (D), and their DNA contents were stained with Hoechst33342 and measured using FACS (E). Click here for file

Additional file 2 **Time-lapse imaging of Fucci2-expressing HeLa cells**. HeLa/Fucci2 cells were grown on a glass-bottom dish and time-lapse imaging was performed using an LCV100 microscope (Olympus). Images were acquired every 15.5 minutes (m). Playback speed is 14,000 × real time. Total imaging time = 96 hours (h). Click here for file

Additional file 3 **Time-lapse imaging of Fucci2-expressing NMuMG cells**. NMuMG/Fucci2 cells were grown on a glass-bottom dish and time-lapse imaging was performed using an LCV100. Images were acquired every 22.5 m. Playback speed is 7,200 × real time. Total imaging time = 50 h. Click here for file

Additional file 4 **Time-lapse imaging of Fucci2-expressing HeLa cells during etoposide treatment**. HeLa/Fucci2 cells were grown on a glass-bottom dish and time-lapse imaging was performed using an LCV100. Cells were treated with 1 μM etoposide. Cells at 4 different positions were imaged every 20.5 m. Playback speed is 7,000 × real time. Total imaging time = 49 h. Most cells were arrested at the G_2 _DNA checkpoint (yellowish green). Click here for file

Additional file 5 **Time-lapse imaging of Fucci2-expressing NMuMG cells during 1 μM etoposide treatment**. NMuMG/Fucci2 cells were grown on a glass-bottom dish and time-lapse imaging was performed using an LCV100. Cells were treated with 1 μM etoposide. Images were acquired every 22.5 m. Playback speed is 7,200 × real time. Total imaging time = 50 h. Most cells were entered M phase and then underwent "nuclear mis-segregation." Click here for file

Additional file 6 **Time-lapse imaging of Fucci2-expressing NMuMG cells during 10 μM etoposide Treatment**. NMuMG/Fucci2 cells were grown on a glass-bottom dish and time-lapse imaging was performed using an LCV100. Cells were treated with 10 μM etoposide. Images were acquired every 22.5 m. Playback speed is 7,200 × real time. Total imaging time = 50 h. The yellowish green-to-red color conversions are indicated by white arrows. Most cells were showed transition from the mitotic to endoreplication cycle. Click here for file

Additional file 7 **Summary of NMuMG/Fucci2 cell responses to etoposide**. Overview diagram of cell populations of normal cycling or G_2 _arrest, nuclear mis-segregation characterized by chromosome fragmentation, tetraploid cells generated by endoreplication, and cell death. Typical fluorescence images of the first three patterns are shown (inset). Scale bars, 10 μm. Click here for file

Additional file 8 **An NMuMG/Fucci2 Cell Approaching 8C**. In the presence of 3 μM etoposide, a cell with a yellowish green nucleus in the normal cycling stayed at G_2 _checkpoint for some time. Then the cell underwent endoreplication to have a red nucleus, which was further converted to a yellowish green one. The yellowish green-to-red color conversion is indicated by an white arrow. No vestiges of mitosis were observed. Scale bar, 10 μm. Click here for file

Additional file 9 **Time-lapse imaging of Fucci2-expressing NMuMG cells during 3 μM etoposide treatment (Additional file **8**)**. NMuMG/Fucci2 cells were grown on a glass-bottom dish and time-lapse imaging was performed using an LCV100. Cells were treated with 3 μM etoposide. Images were acquired every 22.5 m. Playback speed is 15,000 × real time. Total imaging time = 50 h. Only a small fraction of the NMuMG cells were observed to go though multiple endoreplication cycles like the cell featured in this movie. Click here for file

Additional file 10 **An NMuMG/Fucci2 Cell Reaching 8C**. In the presence of 1 μM etoposide, a cell with a yellowish green nucleus underwent endoreplication until 8C DNA content. The yellowish green-to-red color conversions are indicated by white arrows. No vestiges of mitosis were observed. Scale bar, 10 μm. Click here for file

Additional file 11 **Time-lapse imaging of Fucci2-expressing NMuMG cells during 1 μM etoposide treatment (Additional file **10**)**. NMuMG/Fucci2 cells were grown on a glass-bottom dish and time-lapse imaging was performed using an LCV100. Cells were treated with 1 μM etoposide. Images were acquired every 27 m. Playback speed is 16,700 × real time. Total imaging time = 88 h. Only a small fraction of the NMuMG cells were observed to go though multiple endoreplication cycles like the cell featured in this movie. Click here for file
