# Supplementary figures and images for: Drug-induced cell cycle modulation leading to cell-cycle arrest, nuclear mis-segregation, or endoreplication
Source: BMC Cell Biol. 2011 Jan 13;12:2. doi: 10.1186/1471-2121-12-2 (PMC3277280; doi:10.1186/1471-2121-12-2)

A

mCherry-hCdt1(30/120)

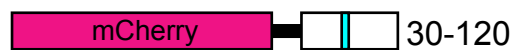

mVenus-hGem(1/110)

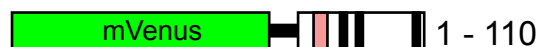

C

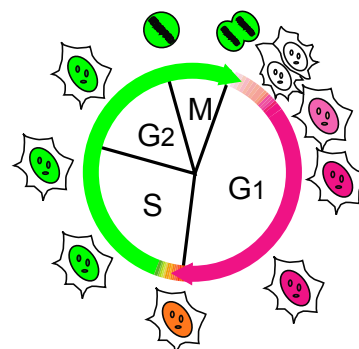

B

Fucci2  
mVenus / mCherry

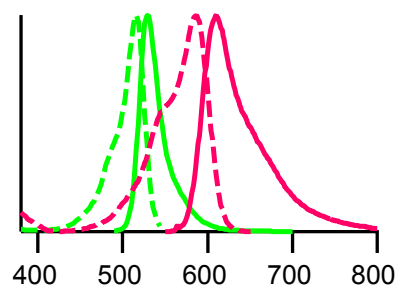

Fucci  
mAG / mKO2

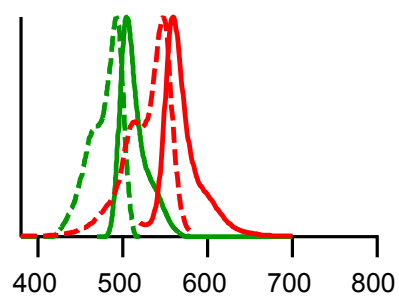

Wavelength (nm)

D

HeLa/Fucci2

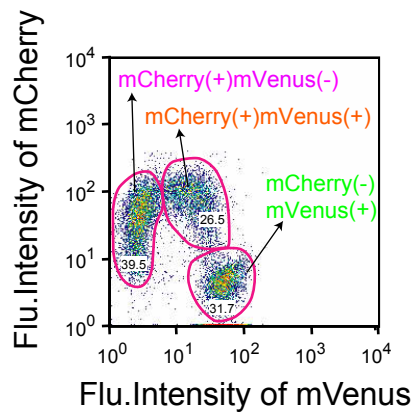

E

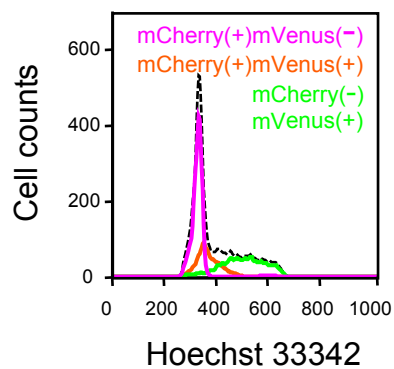

Supplement: Additional file 1 — Construction of Fucci2. (A)Constructs with concatenated mCherry and mVenus fused to deletion mutants of human Cdt1 and Geminin. cyan box, Cy motif; pink box, D (destruction) box; black box, NLS (nuclear localization signal). (B) Excitation (broken line) and emission (solid line) spectra of mVenus and mCherry. (C) Fucci2 labels individual G1 phase nuclei in red and S/G2/M phase nuclei yellowish green. (D,E) Characterization of HeLa/Fucci2 cells. Cells showing red [mCherry(+)mVenus(-)], orange [mCherry(+)mVenus(+)], and yellowish green [mCherry(-)mVenus(+)] fluorescence were collected (D), and their DNA contents were stained with Hoechst33342 and measured using FACS (E). [file 1471-2121-12-2-S1.pdf]

normal  
cell division

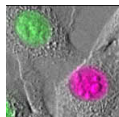

nuclear mis-segregation

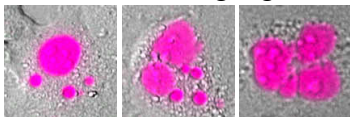

endoreplication

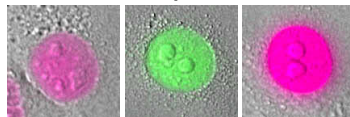

cell death

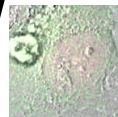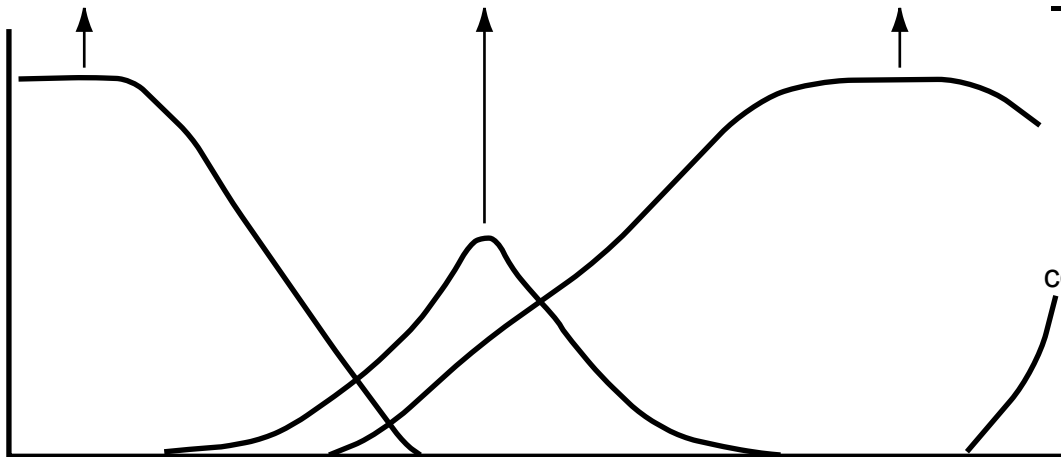

0 0.1 0.3 1 3 10 30 100

etoposide ( $\mu\text{M}$ )

Supplement: Additional file 7 — Summary of NMuMG/Fucci2 cell responses to etoposide. Overview diagram of cell populations of normal cycling or G2 arrest, nuclear mis-segregation characterized by chromosome fragmentation, tetraploid cells generated by endoreplication, and cell death. Typical fluorescence images of the first three patterns are shown (inset). Scale bars, 10 μm. [file 1471-2121-12-2-S7.pdf]

0

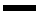

Supplement: Additional file 8 — An NMuMG/Fucci2 Cell Approaching 8C. In the presence of 3 μM etoposide, a cell with a yellowish green nucleus in the normal cycling stayed at G2 checkpoint for some time. Then the cell underwent endoreplication to have a red nucleus, which was further converted to a yellowish green one. The yellowish green-to-red color conversion is indicated by an white arrow. No vestiges of mitosis were observed. Scale bar, 10 μm. [file 1471-2121-12-2-S8.pdf]

NMuMG/Fucci2  
1  $\mu$ M etoposide treatment

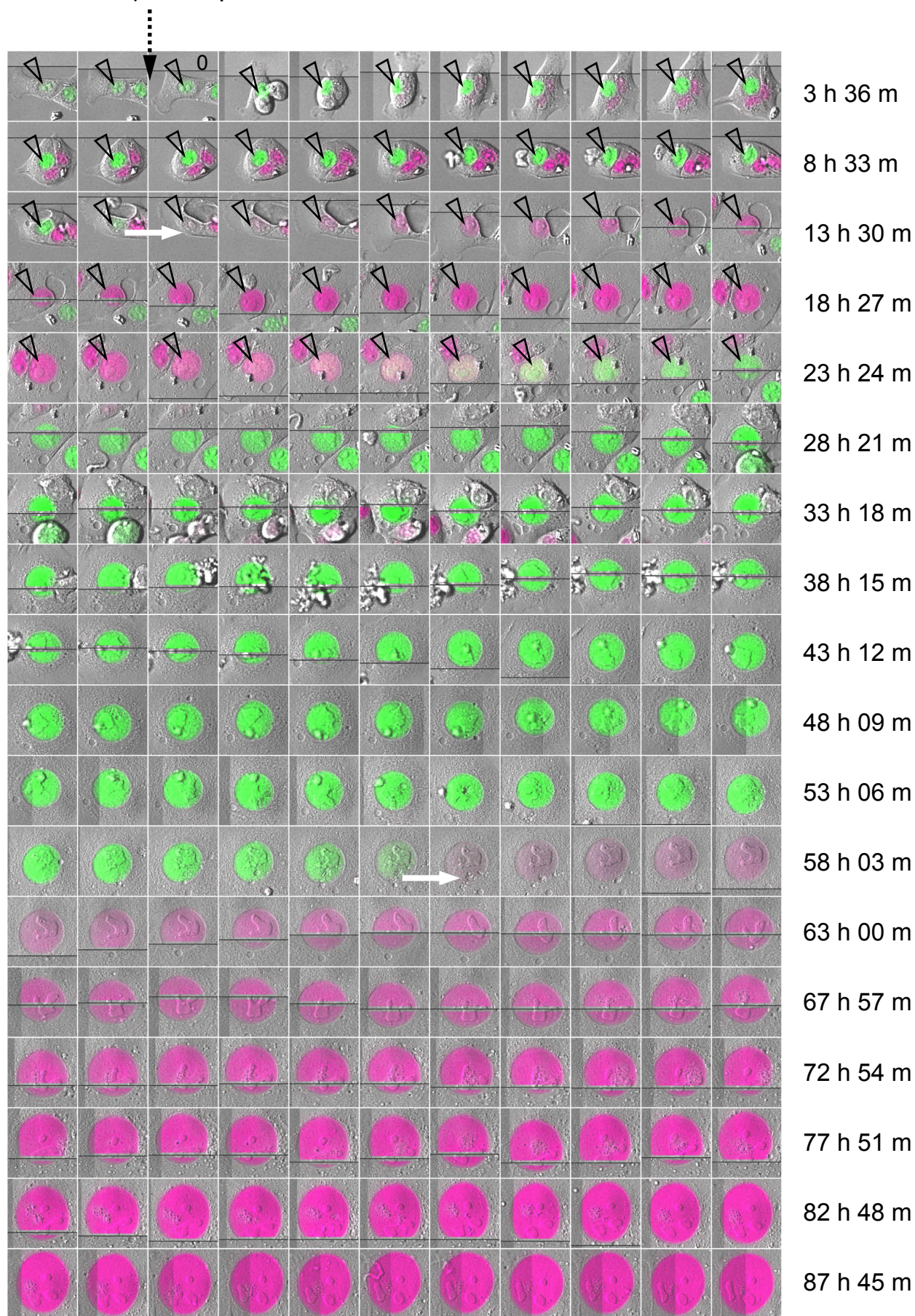

Supplement: Additional file 10 — An NMuMG/Fucci2 Cell Reaching 8C. In the presence of 1 μM etoposide, a cell with a yellowish green nucleus underwent endoreplication until 8C DNA content. The yellowish green-to-red color conversions are indicated by white arrows. No vestiges of mitosis were observed. Scale bar, 10 μm. [file 1471-2121-12-2-S10.pdf]
